# An insight into the epitope-based peptide vaccine design strategy and studies against COVID-19

**DOI:** 10.3906/biy-2006-1

**Published:** 2020-06-21

**Authors:** Murat TOPUZOĞULLARI, Tayfun ACAR, Pelin PELİT ARAYICI, Burcu UÇAR, Erennur UĞUREL, Emrah Şefik ABAMOR, Tülin ARASOĞLU, Dilek TURGUT-BALIK, Serap DERMAN

**Affiliations:** 1 Department of Bioengineering, Faculty of Chemical and Metallurgical Engineering, Yıldız Technical University, İstanbul Turkey; 2 Department of Molecular Biology and Genetics, Faculty of Arts and Science, Yıldız Technical University, İstanbul Turkey

**Keywords:** SARS-CoV-2, COVID-19, epitope-based peptide vaccine design, adjuvant, immunoinformatics

## Abstract

SARS-CoV-2 is a new member of the coronavirus family and caused the pandemic of coronavirus disease 2019 (COVID-19) in 2020. It is crucial to design and produce an effective vaccine for the prevention of rapid transmission and possible deaths wcaused by the disease. Although intensive work and research are being carried out all over the world to develop a vaccine, an effective and approved formulation that can prevent the infection and limit the outbreak has not been announced yet. Among all types of vaccines, epitope-based peptide vaccines outshine with their low-cost production, easy modification in the structure, and safety. In this review, vaccine studies against COVID-19 have been summarized and detailed information about the epitope-based peptide vaccines against COVID-19 has been provided. We have not only compared the peptide vaccine with other types of vaccines but also presented comprehensive literature information about development steps for an effective and protective formulation to give an insight into on-going peptide vaccine studies against SARS-CoV-2.

## 1. Introduction

Coronaviruses belonging to the family Coronaviridae from the members of the order Nidovirales are spherical, enveloped, and single-stranded positive RNA viruses within the diameter range of 60–220 nm, which have rod-shaped glycoprotein extensions in their outer surfaces and carry a genome size of 26–32 kb (King et al., 2011; Li et al., 2020; Shereen et al., 2020). Among the coronaviruses, which are classified into four subgroups: alpha (α), beta (β), gamma (γ), and delta (δ) (Kin et al., 2015), the strains that currently infect humans are seven; HCoV229E, HCoV-OC43, SARS-CoV, HCoV-NL63, HCoV-HKU1, MERS-CoV, and SARS-CoV-2 (severe acute respiratory syndrome coronavirus) (Nomura et al., 2004). Following the severe acute respiratory syndrome (SARS-CoV) that occurred in China in 2002, MERS-CoV caused endemic in the Middle East countries in 2013 (Brian and Baric, 2005), while SARS-CoV-2 created pandemics in 2020^1^1WHO has declared COVID-19 as a pandemic. [online] Website: https://www.who.int/dg/speeches/detail/who-director-general-s-opening-remarks-at-the-media-briefing-on-covid-19---11-march-2020 [accessed date 25.05.2020]. The SARS-CoV-2, which caused the COVID-19 pandemic, belongs to the group of betacoronavirus and its clinical manifestations are evaluated in three different stages: i) mild; weakness, fever, dry cough, fatigue, and upper respiratory tract infections, ii) moderate; shortness of breath, severe cough, diarrhea, iii) severe; severe pneumonia, acute lung injury (ALI), and acute respiratory distress syndrome (ARDS), sepsis and septic shock (Cascella et al., 2020). Currently, there is no specific antiviral therapy developed against SARS-CoV-2. 

Based on previous experiences in SARS-CoV and MERS-CoV outbreaks, some treatment strategies have been developed (Cascella et al., 2020; Mehta et al., 2020; Zhang et al., 2020). These strategies include antiviral treatments or combinations of these that have been known to be safe for humans and used in previous viral outbreaks, which are nafamostat, camostat, chloroquine, imatinib, hydroxychloroquine, remdesivir, favipiravir, lopinavir, ritonavir, etc. (Cascella et al., 2020; Guo et al., 2020).

As of 31 May 2020, 5,891,182 approved cases and 365,966 deaths were reported in more than 100 countries by WHO^2^2Coronavirus Disease (COVID-19) Dashboard. [online] Website: https://covid19.who.int/?gclid=CjwKCAjwq832BRA5EiwACvCWsfv6pvyP1YcbukVtQcwPM_N4d34FlCxLhvTTLJGZGp7WLlh8b5z6bRoCWJsQAvD_BwE [accessed date 31.05.2020]. Vaccine formulations that can be used to protect against high mortality COVID-19 are also currently in the research phase and there are 4 different strategies studied in the clinical stage (those in Phases 1 and 2); i) inactivated or attenuated virus vaccines, a classic method used for viral vaccines (Amanat and Krammer, 2020; Chen et al., 2020), ii) nucleic acid vaccines (DNA and RNA) prepared using the nucleic acid information obtained by rapid sequencing of the entire genome of the virus (mRNA vaccine is the first vaccine to enter clinical phase1 safety trials) (Ma et al., 2020; Saif, 2020), iii) subunit vaccines in which recombinant protein-based vaccines (S protein is the first and main target) are obtained using antigenic proteins of the virus using recombinant DNA technologies and peptide-based vaccines are produced from the epitopes of antigenic proteins of virus (Lu, 2020; Saif, 2020), iv) viral-vector-based vaccines (an alternative option using only S-1 region or receptor binding domain (RBD) to get rid of the problems caused by using all Spike protein) (Ahmed et al., 2020; Amanat and Krammer, 2020; Ma et al., 2020). 

Although epitope-based peptide vaccines are known with their lower immunogenicities compared with other vaccine types which can be increased by utilization of adjuvants, they have become an important subunit vaccine alternative owing to their versatility, low production costs, lower allergenic and reactogenic responses. Therefore, developing an epitope-based peptide vaccine against SARS-CoV-2 along with other vaccine alternatives is of great importance. In this review, while the stages of detection, development, production, and biological evaluation of peptides that can be used in vaccine formulations against COVID-19 will be explained in detail, the difficulties experienced in the development of epitope-based peptide vaccines, and the advantages and disadvantages of these vaccines will also be discussed.

## 2. Vaccine design strategies against SARS-CoV-2 

The development of vaccine against SARS-CoV-2 is one of the most important strategies to prevent COVID-19 and control the outbreak. Today, various biotechnology companies/universities are putting great efforts in vaccine development studies. According to the WHO data of May 29th, 2020, 10 candidate vaccines based on nonreplicating viral vector, DNA, inactivated virus, RNA, nonreplicating viral vector, and protein subunit are in clinical evaluation and 121 candidate vaccines based on different technologies are in preclinical evaluation against COVID-19. 

Among the on-going preclinical vaccine candidate studies, there are 10 DNA, 5 inactivated viruses, 3 live attenuated viruses, 15 nonreplicating viral vectors, 33 subunit proteins, 15 replicating viral vectors, 16 RNA, and 13 virus-like particles. Eleven of the preclinical studies about COVID-19 vaccine are on peptide-based vaccine candidates^3^3COVID-19 vaccines. [online] Website: https://www.who.int/who-documents-detail/draft-landscape-of-covid-19-candidate-vaccines [accessed 31.05.2020].

Different vaccine candidates have several advantages and disadvantages compared to each other (Ozkan, 2020; Shang et al., 2020). However, it has been stated that the most important features expected in the developed vaccine candidate are long-term protection, antigen-specific cellular immunity, and ability to induce the systemic immune system (Ozkan, 2020). 

### 2.1. Inactivated virus vaccines

Inactivated virus vaccines consist of virus particles produced in culture that have lost the ability to cause disease (Zhang et al., 2020). The virus is killed by using heat, UV irradiation, or chemicals (formaldehyde etc.) to reduce virulence, so that a vaccine-induced infection can be prevented. Large-scale cultures of SARS-CoV-2 were created and inactivated viruses were obtained from these cultures by researchers via known methods (Zhang et al., 2020). 

Although inactivated virus vaccines have advantages such as safety, easier preparation, and inducing high-titer neutralizing antibodies, it is an important disadvantage that they are potentially inappropriate for highly immunosuppressed patients (Roper and Rehm 2009; Shang et al., 2020). Additionally, preparing the inactivated virus vaccine requires large quantities of infectious virus (Callaway, 2020). Recently, Sinovac Biotech and their consortium have produced a vaccine candidate from purified inactivated SARS-CoV-2 viruses. It was shown in the study that specific antibodies against the SARS-CoV-2 were detected in 3 different animals which had been immunized with the inactivated virus vaccine candidate (Gao et al., 2020).

### 2.2. Attenuated virus vaccines

Attenuated vaccines are prepared by mutating the virus as a result of a serial treatment with human or animal cells in the laboratory to decrease its ability to cause disease. One of the most important advantages of attenuated vaccines is that they offer more than one antigenic component to the host, so it can induce various immunological effectors against the virus (Zhang et al., 2020). It has been reported that many research institutions/universities such as The Chinese Centers for Disease Control and Prevention, Wuhan Institute of Virology, Chinese Academy of Sciences, Zhejiang University, Codagenix Inc., and Serum Institute of India, Ltd. are carrying out attenuated vaccine studies (Zhang et al., 2020). For example, Codagenix continues its vaccine development studies against SARS-CoV-2 by using their own codon deoptimization technology.

### 2.3. Subunit vaccines

Subunit vaccines consisting of purified antigens have the advantages of easy production, safety, and the ability to induce both cellular and humoral immune response (Roper and Rehm, 2009; Shang et al., 2020). However, subunit vaccines may require adjuvants (immunostimulatory molecules delivered alongside the vaccine) and multiple doses to increase their immunogenicity and protective activity (Callaway, 2020). CoV S proteins, which have been shown in studies that have the ability to generate protective antibodies, have been identified as favorite targets in subunit vaccine development against SARS-CoV (Enjuanes et al., 1995; Navas-Martin and Weiss, 2003). In studies, with similar subunit vaccines, against SARS-CoV, it is known that experimental animals are protected from infection, although these vaccine candidates have not been tested in humans yet (Callaway, 2020).

Similarly, in various studies, it has been accepted that S1 protein and/or RBD element of SARS-CoV-2 should be considered the foremost antigen in vaccine development studies against COVID-19 (Callaway, 2020; Lon et al., 2020; Rogers et al., 2020; Shang et al., 2020; Tang et al., 2020). Many research teams including biotechnology companies, universities, research centers, etc. are working on effective vaccines containing the viral protein subunits against COVID-19 (Lon et al., 2020; Rogers et al., 2020; Tang et al., 2020).

### 2.4. Viral vector vaccines

Viral vector vaccines are prepared by inserting the DNA fragment of the highly infected virus into another harmless virus. One of the advantages of this vaccine type is that it can induce high cellular and humoral immune responses (Shang et al., 2020). It has also been reported that while viral vector vaccines can be safe similar to subunit vaccines, the immunogenicity of viral vector vaccines can be highly analogous to live attenuated vaccines (Wu, 2020). 

University of Oxford and the US National Institutes of Health (NIH) collaborators produced an adenovirus expressing the SARS-CoV-2 S protein (van Doremalen et al., 2020). The study conducted in mice and rhesus macaques showed that this vaccine candidate induced immune response against SARS-CoV-2. Additionally, the vaccine efficiency study showed that the viral load decreased, the lung of vaccinated animals had no signs of virus replication, and no lung damage was observed in vaccinated animals compared to control (van Doremalen et al., 2020). 

### 2.5. mRNA vaccines

mRNA vaccines, which have been previously investigated for different diseases and had successful results, are easier to design and translate into clinical trials (Ozkan, 2020; Roper and Rehm, 2009; Shang et al., 2020). Although mRNA vaccines have advantages such as easier design and inducing strong immune response, it is an important disadvantage that they are extremely unstable under physiological conditions (Rauch et al., 2018). Moderna and a consortium consisting of Fudan University/Shanghai Jiaotong University/Bluebird Biopharmaceutical Company carry out their studies on mRNA vaccines against COVID-19. While Moderna’s mRNA vaccine encode S protein, the consortium is working on two different mRNA vaccines that express S protein and virus-like particles (Zhang et al., 2020). Apart from these, it has been reported in the literature that various companies/universities and research centers are also working on mRNA vaccines (Zhang et al., 2020).

### 2.6. DNA vaccines

In DNA vaccines, antigen or antigens are expressed by the plasmid DNAs. The difference from mRNA vaccines is the coding of antigens with plasmid DNA. Although they are more stable than mRNA vaccines, are easy to design, and have the ability to form highly neutralized antibodies, it has been reported that repeat doses of DNA vaccine may cause toxicity (Rauch et al., 2018). 

In the study of the USA and Israel research teams, DNA-based vaccines have been developed to express S protein of SARS-CoV-2 in different forms (Yu et al., 2020). In the study, both humoral and cellular immune responses have been developed in the animals immunized with the DNA vaccine. Additionally, the virus challenge study showed that the vaccine encoding full-length S protein decreased the viral load in bronchoalveolar lavage and nasal mucosa was decreased in comparison with the control group (Yu et al., 2020). 

### 2.7. Epitope-based peptide vaccines

As outlined above, traditional vaccines against COVID-19 include live/attenuated/inactivated SARS-CoV-2. However, there are lots of disadvantages to the use of whole-organism vaccine especially in immunosuppressed patients if there is a presence of immunologically redundant biological components or biological impurities (Roper and Rehm, 2009; Shang et al., 2020). Thus, subunit vaccines have become widespread especially in research area due to their safety profiles and easier production. Although proteins are the main type of subunit vaccines, peptides, which are small fragments of proteins, are also used as subunit vaccines (Callaway, 2020).

Epitope-based peptide vaccines are used to overcome the problems of possible side effects related to heterogeneous and multicomponent vaccine and are considered an alternative to conventional vaccines. Peptides used in the vaccines are synthesized chemically using the amino acid sequences of the antigenic proteins of targeted pathogens. These vaccines contain antigenic peptide epitopes with 20–30 amino acids having high immunogenicity. The recognition of the antigenic peptides by the immune system triggers the immune response by stimulating cytotoxic T cells or B cells, or both simultaneously (Moisa and Kolesanova, 2012). Although epitope-based peptide vaccines significantly limit allergenic or reactogenic complications, low molecular weight, and easy clearance from the body make the peptides lower immunogenic which can be overcome by using carriers or adjuvants in peptide vaccine formulations to increase their immunogenicity and decrease the dose (Moisa and Kolesanova, 2012).

Epitope-based peptide vaccines have many advantages over other vaccines. It is easier and cheaper to produce peptides compared to traditional vaccines. Epitope-based peptide vaccines can be standardized and batch-to-batch differences can be very low. Since the structure of the peptide is very well-known, structure-function relationships can be correlated much easier than the conventional vaccines. Also, the structure of the peptide can easily be modified to obtain multiepitope or conjugated structures (Moisa and Kolesanova 2012; Sesardic 1993).

## 3. Stages in development of epitope-based peptide vaccines

Although an epitope-based peptide vaccine formulation basically contains the antigen and the adjuvant, development of the vaccine should follow a pathway to prove its effect and protection against the disease. The first stage is to design the amino acid sequence of antigenic peptide from the proteins of pathogen. Although experimental techniques and in silico methods are used together to determine the epitope, in silico methods have become prevalent for prediction of the epitopes due to their time and cost-effective nature compared to experimental techniques (Li et al., 2014; Sanchez-Trincado et al., 2017). In the second stage, peptide should be produced with high purity based on chemical and biological methods in which chemical synthesis of peptide has been the routine method especially for the peptides having less than 50 amino acids (Behrendt et al., 2016). An appropriate adjuvant selection is the third stage for mixing or conjugating with the peptide to augment the immune response against the peptide (Yang and Kim, 2015). Adjuvant used in the epitope-based peptide vaccine can modulate the interactions of peptide with different cells of immune system (Guy 2007). The last stage is the evaluation of vaccine formulation in vitro and in vivo to determine the immune response against the peptide (Mozdzanowska et al., 2003). It is important to comprehend the working mechanism of the vaccine formulation to relate the structure with its interactions with immune system cells. However, in vivo and in vitro evaluation of the epitope-based peptide vaccine gives limited information without the challenge studies against the pathogen (Swee et al., 2013). Therefore, if sufficient immune response is obtained from in vivo and in vitro studies, vaccine formulation should be challenged against the pathogen to reveal the protection efficiency before preclinical and clinical studies (Swee et al., 2013; Zhao et al., 2010).

### 3.1. Design of epitope-based peptide sequences by in silico analyses 

The immune system is a large network that interacts with each other and contains thousands of molecular networks with the responses it creates (Tomar and De, 2010). Genomic sequencing, clinical practice, and large amounts of accumulated data generated by epidemiological data may cause confusion. In order to use this large data more effectively by scientists, it must be stored, managed, and analyzed (Bahrami et al., 2019). This situation has brought immunology and computer sciences together and formed immunoinformatics (Pourseif et al., 2019; Tomar and De, 2014). 

Immunoinformatics is a new and important area with a large database that can create the most suitable options before the experimental stage (Bahrami et al., 2019). Immunoinformatics includes an in silico approach, which is a more stable and less costly method in a shorter period of time to conventional laboratory techniques for development of vaccines (Ali et al., 2017; Khan et al., 2019). The development of vaccines using conventional techniques entails high cost and a long period of 10–15 years (Parvizpour et al., 2020). With this approach, specific regions are designed to prevent unwanted immune responses, long-term immune responses to required responses, and cost–time effectiveness (Parvizpour et al., 2020). The first step in the in silico-approached immunoinformatic epitope-based peptide studies is to identify the virus’s antigen that stimulates antibodies and targets that are important for immunity (Kibria et al., 2020). The structural proteins of Spike (S), Envelope (E), Membrane (M) and Nucleocapsid (N) encoded by SARS-CoV, MERS-CoV, and SARS-CoV-2 genomes belonging to the betacoronavirus genus are suitable candidates for antigen selection (Ahmed et al., 2020). Since the B and T cell epitopes, an immunogenic site of an antigen, produce humoral and cellular immune responses, these regions are predicted (Parvizpour et al., 2020). 

Different web-based tools are available for epitope design (Bui et al., 2007). These tools provide user convenience by combining the methods developed for the determination of epitopes. The Kolaskar and Tongaonkar method is a semiexperimental method that uses the physicochemical properties of amino acids and the experimentally determined data of these amino acids in the B cell epitope design (Kolaskar and Tongaonkar, 1990). The BepiPred-2.0 server estimates B cell epitopes on only crystal structure derived data, hence providing higher quality and strong prediction power (Jespersen et al., 2017). Basically, the methods used in B cell epitope estimation is based on parameters such as the flexibility, hydrophilicity, polarity, exposed surfaces, antigenic propensity, and accessibility of peptide chains (Vita et al., 2019). The method used for T cell epitope prediction generally uses MHC class 1 binding, TAP transport efficiency, and proteasal processing algorithms (Kibria et al., 2020; Vita et al., 2019). T cell epitope prediction approach takes advantage of the cross-reactivity between HLA class 2 regions and alleles to predict strong epitopes without being affected by HLA polymorphism and ethnicity (Grifoni et al., 2020). Then, population-based T epitope estimation is made and distribution of MHC alleles in a defined population is examined. With this estimated population coverage, the percentage of potential individuals who will be immune to the identified T cell epitope is revealed (Ahmed et al., 2020). These epitopes are then transformed into 3D structure and docking analysis performed to show the interaction of the determined epitopes with different HLAs (Oany et al., 2014). Another stage is the allergenicity test. This step is vital for predicting the allergic reactions that may develop after giving the vaccine into the body (Oany et al., 2014).

Epitope-based peptide vaccine candidates have been identified in many studies against MERS-CoV, SARS-CoV, and SARS-CoV-2, using all these in silico approaches. Although an approved vaccine is not available yet for MERS-CoV seen for the first time in 2012 (Yong et al., 2019), protein-based studies at the preclinical level are quite high (Cho et al., 2018). In these studies, a large number of possible epitopes have been identified using in silico methods (Badawi et al., 2016; Ibrahim and Kafi, 2020; Shi et al., 2015). Similarly, a large number of vaccine development studies using in silico approach have also been conducted for SARS-CoV (Wang et al., 2004; Ying et al., 2003), which emerged in 2003 and spread to 26 countries and with more than 8000 cases. Almofti et al. reported that 3 B cell epitopes and 5 T cell epitopes, which gave the best scores, could be very good vaccine candidates determined by in silico-based epitope design study using NCBI and IEDB tools (Almofti et al., 2018). Vaccine development studies has also been conducted rapidly against SARS-CoV-2 (Le et al., 2020), as the COVID-19 outbreak announced as a pandemic. Within the immunoinformatic approach, many studies reported B and T cell epitopes and proposed as potential vaccine candidates for SARS-CoV-2 (Baruah and Bose, 2020; Joshi et al., 2020; Kiyotani et al., 2020). An in silico-based study conducted by Kalita et al. identified 33 nontoxic, nonallergenic, thermostable epitopes from 3 proteins (Kalita et al., 2020). The stage of vaccine development studies initiated against COVID-19 is given in detail in Section 3.3.

In the light of all these studies and data, immunoinformatics offers strong candidates that could be used in new vaccine designs (Sobolev et al., 2005). Many different databases and pieces of software for vaccine design and validation (Parvizpour et al., 2020) are now available for prediction of epitope-based peptides fast and accurate with possible strong immunity and minimal side effects (Parvizpour et al., 2020). Further in vitro and in vivo tests should be conducted on B and T cell epitope-based peptides predicted by in silico approaches following their synthesis. 

### 3.2. Peptide synthesis, characterization and purification

Generally, peptides can be chemically synthesized using two strategies: solution- and solid-phase synthesis (Jensen et al., 2013). The most useful and popular strategy is the solid-phase method named as Merrifield solid-phase peptide synthesis (SPPS) developed by R. B. Merrifield (Merrifield, 1963). 

There are two main types of solid-phase peptide synthesis derived from the name of the N-terminal protecting groups of the amino acids that determine the chemistry method to be used: Boc and Fmoc chemistry. Since removing the Boc protecting group requires working with very strong acids such as hydrogen fluoride (HF), the Fmoc chemistry is preferred. SPPS with Fmoc chemistry can be performed under less extreme conditions than Boc chemistry, and removing of the side protecting groups, and cleaving of the peptide from the resin (solid-phase) can occur (Chan and White, 1999). Here the resin consisting of cross-linked polymeric beads has swelling property in solvents such as DMF and DCM and contains various linker groups on it that binds amino acids. The C-terminal amino acid of the peptide binds to these resin beads. The peptide is constructed with stepwise addition of amino acids at the N-terminus. At the end of the synthesis, the peptide is cleaved from the resin as the side chain protecting groups of amino acids are removed simultaneously (Stawikowski and Fields, 2012). 

The peptide needs to be characterized after its synthesis. LC-ESI-MS, MALDI-TOF-MS, and Q-TOF-MS methods are used extensively for molecular weight determination of the synthesized peptide. LC-ESI-MS and Q-TOF-MS systems are frequently used in routine as they also allow method validation, determination of purity, and method development for the purification procedure (Acar et al., 2019). While FT-IR spectroscopy is used to determine the functional group characteristics of the peptide, two- and three-dimensional structure of the peptide is illuminated by circular dichroism spectroscopy. In addition, measurement methods such as UV-VIS fluorescence spectrometry are also used for further characterization (Ucar et al., 2019). 

The peptide should then be separated from the impurities by proper purification techniques. The reversed phase preparative HPLC is the mostly used technique for purification of peptides. Ion exchange and affinity chromatography can also be used during purification of the special peptides after production (Ucar et al., 2019).

### 3.3. Peptide sequences for epitope-based peptide vaccine studies against SARS-CoV-2

For the vaccine against COVID-19, the studies in which antigenic peptides are chemically synthesized, and/or designed with in silico methods and showing possible antigenic activities are detailed below.

Peptide epitope-based vaccine development studies are focused on S protein. Studies for the determination of the right peptide sequence for a vaccine against COVID-19 are based on in silico studies, synthesis, and isolation of antigenic peptides. In this part of the review, information is given about peptide sequences that can be used against SARS-CoV-2.

In a study by Fast et al., it was revealed that T cell and B cell epitopes identified in the study can be used in vaccine formulations against SARS-CoV-2 with higher immune responses and in detection of neutralizing antibodies (Fast and Chen, 2020). The study reported that the 494-506 sequence (SYGFQPTNGVGYQ) of S protein has the highest MHC presentation and is a B cell epitope. It is stated that the data obtained from the study is important for identifying strong epitope-based vaccine candidates, as well as its interaction with clinical results will provide information about the relationship between antigen presentation scores and vaccine efficacy (Fast and Chen, 2020). 

Bojin et al. identified a multi-epitope peptide vaccine candidate against SARS-CoV-2, which could have the potential to stimulate T cell immune responses (Bojin et al., 2020). Short epitopes with the capacity to bind to multiple HLA alleles were selected. MHC class-I and MHC class-II restricted epitopes were attached with a cathepsin-sensitive linker peptide (LLSVGG). A long synthetic peptide sequence has been identified as SYFIASFRLFARTRSLLSVGGYLQPRTFLL [A*0201 allele (MHC-I epitop)-cathepsin-sensitive linker-DRB1*1101 allele to be used in vaccine candidates (MHC-II epitop)] (Bojin et al., 2020). 

In a study by Li et al. interactions of SARS-CoV-2 with B- and T-cell epitopes and human HLA alleles for surface glycoprotein were studied (Li et al., 2020). It was indicated that the VGYQPYRVVVLSFEL (MHC-II epitope) peptide sequence had the highest antigenicity score.

In another investigation that examined antigenic peptide sequences of SARS-CoV-2 with multiple epitope structure, proteins of S, E, and M were studied (Feng et al., 2020). While 25 sequences of S protein are recommended, 2 sequences have been determined for M and E proteins. Of all these peptide sequences, the 1216-1245 sequence (IWLGFIAGLIAIVMVTIMLCKKKKKKKKK) of S protein gave higher HLA scores than others and was proposed as a candidate antigen for use in vaccine formulations (Feng et al., 2020). 

Zhang et al. have prepared SBP1 peptide-based linker with 23 amino acids using molecular dynamic (MD) simulations in their study (Zhang et al., 2020). The human protein derivative SBP1 sequence is completely proteinogenic and is not considered to have an immunogenic effect. It is seen that S protein is targeted in most of the drug and vaccine development studies against SARS-CoV-2. The S protein consists of two subunits, S1 and S2. The S1 has an RBD that interacts with the receptor of host cell, ACE2. This peptide linker is thought to inhibit the interaction between S protein of SARS-CoV-2 and ACE2, prevent virus entry into human cells, and play an important role in diagnosis and treatment of SARS-CoV-2 (Zhang et al., 2020). 

Pant et al. have published a study providing comprehensive research of main residues and ligand-receptor interactions for the improvement of peptide-like molecules as COVID-19 essential protease inhibitors (Pant et al., 2020). They reported that four small molecules selected from 300 peptide-like molecules found in various databases, with docking studies and MD simulations, show powerful binding affinity with residues within the binding position and should be evaluated against SARS-CoV-2 (Pant et al., 2020). 

In one study, the authors presented in silico description of an exhaustive list of antigenic peptides that could potentially be used to develop vaccines against the new coronavirus 2019 (Lee and Koohy, 2020). They conducted de novo investigation of peptides with 9 amino acids from SARS-CoV-2 binding to HLA alleles in the European and Chinese populations. Also, the peptides had TCR recognition potential determined by the immunogenicity algorithm of iPred developed recently (Lee and Koohy, 2020). 

The ITLCFTLKR epitope was chosen depending on its good antigenicity to prepare and design a vaccine against COVID-19 (Joshi et al., 2020). It was reported that this particular epitope can actively bind to MHC HLA-Alleles and covers the maximum population for various geographic districts. However, it was indicated that the use of this particular peptide epitope in the design of vaccines against SARS-CoV-2 requires wet laboratory validation. 

Peptides having more than one epitope can be designed and used in the vaccines targeting the S protein. In a study conducted by Bhattacharya et al., thirty-three linear B cell epitopes have been determined from the S glycoprotein of SARS-CoV-2 (Bhattacharya et al., 2020). The sequences were then analyzed to determine the T cell epitope that can be combined with MHC-I and II. The selected 16 epitopes were transformed into a single sequence with (EAAAK)3 linkers. Then, the peptide-based vaccine composition was designed and the model was confirmed to be of good quality. Molecular docking between the designed multiepitope peptide and TLR-5 exhibited a significant binding affinity. This proves the importance of designing multiepitope peptides to develop effective vaccine candidates against COVID-19. These epitopes are ideal candidates for formulating a multiepitope peptide vaccine when in vitro and in vivo validation is performed, owing to their B-cell epitope containing sequences and verified antigenicities (Bhattacharya et al., 2020). On the other hand, Abdelmageed et al. suggested that it can be generated a strong and long-term immune response against the COVID-19 by CD8 T cell to produce an immunogenic peptide vaccine (Abdelmageed et al., 2020). Therefore, they studied T cell epitope-based peptide vaccine modeling instead of B cell by targeting E protein. In MHC-I for the peptide vaccine, the following 10 peptide sequences (YVYSRVKNL, SLVKPSFYV, SVLLFLAFV, FLAFVVFLL, VLLFLAFVV, RLCAYCCNI, FVSEETGTL, LTALRLCAY, LVKPSFYVY, and NIVNVSLVK) were selected as the best candidates with the highest world population coverage (88.5%). For MHC-II, another 10 peptide sequences (KPSFYVYSRVKNLNS, VKPSFYVYSRVKNLN, LVKPSFYVYSRVKNL, PSFYVYSRVKNLNSS, NIVNVSLVKPSFYVY, LLVTLAILTALRLCA, SFYVYSRVKNLNSSR, LVTLAILTALRLCAY, VTLAILTALRLCAYC, and CNIVNVSLVKPSFYV) were also selected as vaccine candidates according to the world population coverage (99.99%). The research group, which aims to find the protected coding genes necessary for the survival of the virus, suggested that unique genes can be used as a potential drug and vaccine target due to their large mutations that enable the virus to show high resistance to antibiotics. Protein E has been identified as the most antigenic gene, and its protection against 7 coronavirus strains has been tested, validated, and multiple sequence alignment was done (MSA) (Abdelmageed et al., 2020).

As a result of another detailed study conducted with a computer-aided vaccine design (CAVD) approach to develop a series of epitope-based peptide vaccine candidates, 4 possible peptides were presented; PELDSFKEELDKYFKNHTSPDVDLGDISGIN (1143-1173), FSQILPDPSKPSKRSFI (802-818), TMSLGAENSVAYSNNS (696-711), and NSNNLDSKVGGNYN (437-450) (Biswas et al., 2020). Researchers stated that if the peptide sequences are supported by wet laboratory studies, they may be candidates in developing vaccines against COVID-19. 

Grifoni et al., in their study, emphasized that understanding of SARS-CoV-2 proteins and epitopes recognized through human CD4+ and CD8+ T cells responses are important in developing vaccines because it will allow monitoring, measuring, and understanding immune responses against COVID-19 infection (Grifoni et al., 1920). It was stated that this information would assist candidate vaccine modeling and facilitate the assessment of vaccine immunogenicity. Using the peptide “megapooller” prescribed by HLA class I and II, the research group detected CD8+ and CD4+ T cells specific to SARS-CoV-2 in the blood samples of 70–100% of healing COVID-19 patients. According to the results, 15-mer peptides were synthesized to investigate spike-specific CD4+ T cells. Using multiple experimental approaches, CD4+ and CD8+ T cell and antibody responses specific to SARS-CoV-2 were observed through all COVID-19 cases. Besides, SARS-CoV-2-cross-reactive T cell responses were observed in healthy donors, demonstrating some potential for preexisting immunity in the human population (Grifoni et al., 1920).

### 3.4. Adjuvants used in epitope-based peptide vaccines

Peptides have lower immunogenicity compared to conventional attenuated or inactivated vaccines due to their low molecular weight and size, susceptibility to degradation and lack of all antigenic sites available in the pathogen. Therefore, additional molecules called adjuvants should be used in the peptide vaccines to increase the immune response. Adjuvants are used especially in subunit vaccines to enhance and modulate the immune response against the antigen, and delivery and presentation of the antigen to antigen presenting cells (APC) (Khong and Overwijk, 2016). Among several immunological roles of adjuvants in vaccine formulations (Powell et al., 2015), increasing the antibody titer, decreasing the antigen dose, inducing the potent cell-mediated immunity, and expression of costimulatory molecules and cytokines are the most important properties of adjuvants (Khong and Overwijk, 2016). 

Adjuvants used in epitope-based peptide vaccines can be simply classified into two main classes as immunostimulants for increasing the immune response and vehicles for delivery and controlled release of antigens to the immune system (Reed et al., 2009). Although various adjuvants can be used in vaccine formulations, adjuvants used or proposed for the peptide and subunit vaccines against SARS-CoV-2 and related viruses will be discussed to remain within the scope of the review.

#### 3.4.1. Alum 

Alum has been the most commonly used adjuvant in vaccines since their discovery in by Glenny et al. (1926) and classified in branch of vehicles of adjuvants. In the literature, alum is generally used to name aluminum hydroxide and aluminum phosphate gels as adjuvant. In the study by Tseng et al. (2012), alum was used as the adjuvant in four different vaccine formulations against SARS-CoV, in which two of them are whole virus vaccines, one of them is recombinant S protein vaccine, and the last one is virus-like particle. These vaccine formulations were examined with and without alum as the adjuvant. As mentioned in the study, almost all formulations protected the mice against the virus but Th2-type immunopathology and hypersensitivity was observed in response to the formulations. No direct relationship could be established between alum usage as the adjuvant and hypersensitivity. Jaume et al. developed a subunit vaccine formulation against SARS-CoV using recombinant S protein with and without alum as the adjuvant (Kam et al., 2007). They investigated immunogenicity of the vaccine and antibody-dependent enhancement (ADE) on hamster in their study, in which viral load in hamsters at 3 days postchallenge decreased by at least four orders of magnitude without signs of enhanced lung pathology. Unfortunately, alum cannot induce Th1 immune response effectively, which is significant in vaccines against viruses (Petrovsky and Aguilar, 2004).

In a novel study, aluminum nanoparticles were used as antigen delivery vehicles (adjuvants) to deliver ovalbumin as the model subunit antigen through pulmonary administration which is not suitable for conventional alum (Wang et al., 2020). It was shown that aluminum nanoparticles can induce both Th1 and Th2 responses safely and were effective against air-borne pathogens like MERS or SARS-CoV-2 because of the pulmonary administration of the vaccine formulation. 

#### 3.4.2. AS03

In ongoing studies against SARS-CoV-2, GlaxoSmithKline is using the adjuvants of AS03 in their cooperative study with Clover Biotechnology for developing S protein based subunit vaccine (Veugelers and Zachmann, 2020). AS03 is an oil-in-water emulsion-based and vehicle type adjuvant that contains squalene and alpha-tocopherol (Reed et al., 2009). Usage of this adjuvant induces both Th1 and Th2 cytokine responses and higher antibody titer compared with alum as shown in the vaccine study against Hepatitis B (Morel et al., 2011).

#### 3.4.3. Emulsigen

Khurana et al. studied S protein-based vaccines against SARS-CoV-2 to evaluate induced antibody titers (Ravichandran et al., 2020). They used Emulsigen as the adjuvant in their vaccine formulations. Emulsigen is another oil-in-water emulsion-based adjuvant system. The study revealed that more antibody titers were obtained against receptor binding domain and S1 protein than the S2 domain.

#### 3.4.4. MPLA and CpG

MPLA and CpG were used as adjuvants in a liposomal vaccine formulation against SARS-CoV-2 that contains S protein as the antigen (Lixin et al., 2020). While both CpG and MPLA (monophosphoryl lipid A) are immunostimulant type adjuvants, CpG has a nucleic acid-based structure and MPLA is a lipid. The adjuvant system in the study induced a humoral and CD8+ and CD4+ T-cell responses against S protein of SARS-CoV-2.

#### 3.4.5. Matrix-M™ 

The company of Novavax is also developing a subunit vaccine against SARS-CoV-2 using antigenic proteins of the virus (Veugelers and Zachmann, 2020). The company uses their commercial adjuvant of Matrix-M™ in the vaccine to increase and modulate the immune response. It is known from previous studies that Matrix-M™ is a nanoparticle that is formed from Quillaja saponins with cholesterol and phospholipids and preferentially induces TH1 immune response against the antigenic molecules (Magnusson et al., 2018).

#### 3.4.6. CpG ODN, polyI:C (polyinosinic:polycytidylic acid) and R848

Zhao et al. compared the immune responses obtained from three different adjuvants used in peptide vaccines against SARS-CoV (Zhao et al., 2011). In the study, CpG ODN, polyI:C (polyinosinic:polycytidylic acid) and R848 were examined as immunostimulant adjuvants. CpG ODN had better immune response than the others owing to induction of IL-12 by CpG. Also, the authors noticed that these three adjuvants not only induce the cellular immune response, but also induce humoral immune response against the virus by using the peptides as the antigens. 

#### 3.4.7. cGAMP

Cyclic guanosine monophosphate–adenosine monophosphate (cGAMP) was also proposed to be used as adjuvant in SARS-CoV-2 vaccine in the form of PS-GAMP (pulmonary surfactant GAMP) (Ma et al., 2020). This adjuvant can be a good “universal” mucosal adjuvant to develop a vaccine against a wide range of viruses owing to its broad-spectrum cross protection not only against H1N1 but also against H3N2, H5N1, and H7N9 viruses.

#### 3.4.8. Liposomes 

Matsui et al. examined a different strategy by attaching the synthetic antigenic peptides of SARS-CoV onto the surface of liposomes to induce immune response against the virus (Ohno et al., 2009). Liposomes are a kind of vehicle or delivery type of adjuvants. In the study, one of the formulations of antigenic peptide coated liposomes exhibited protection in viral challenge. 

There are several on-going subunit vaccine studies that use adjuvants to induce the immune response and, as mentioned, adjuvant type can cause different forms of immune responses against the antigens and pathogen. Therefore, it is important to notice that proper adjuvant selection becomes vital especially in SARS-CoV-2 vaccines to prevent immunopathology and ADE. 

### 3.5. Immune response against SARS-CoV-2 infections

In order to design and develop the appropriate and effective vaccine against SARS-CoV-2, the pathogenesis of the infection must be known well before the vaccine development study. Coronaviruses bind to the cell membrane by using the S protein, which is very spiky shaped, to infect a cell (Callaway, 2020; Rogers et al., 2020; Shang et al., 2020). Similar to SARS-CoV, the SARS-CoV-2 binds the ACE2 receptor on the cell surface for infection in humans (Lon et al., 2020; Rogers et al., 2020; Tang et al., 2020). During CoV infection in humans, the entry of double chain viral RNA occurs inside the double membrane vesicles. Antigen-presenting cells (APC) as an antiviral mechanism act in the antigenic peptide presentation in complexes with MHC-I and MHC-II to CD4+ and CD8+ T cells and one of the most important cells is dendritic cells (DC) (Kumar et al., 2020).

As with any viral infection, dendritic cells (DC) present in the respiratory system play an important role as antigen presenting cells (APC) during SARS infection. Antigen presentation in SARS-CoV is importantly done with MHC-I and then MHC-II and they are also investigated for epitope mapping used generally for vaccine development. In the studies, it has been identified that approximately 405 T cell peptide epitopes have a high affinity against MHC-I and MHC-II. And also, two different B cell epitopes, which are strong neutralizing, were found for the S protein (Kumar et al., 2020).

Cellular immune response occurs in infected cells through T-lymphocytes as a mechanism of adaptive immunity. While T helper cells are active in the general adaptive immune response, Tc (cytotoxic T) cells are responsible for the clearance of the virus-infected cells. It has been demonstrated that CD4+ (TNFa, IL-2, and IFN) and CD8+ (TNFa, IFNγ) memory T cells can function by replicating the IFNγ-producing T cell. However, in recent reports on SARS-CoV-2, it was suggested that an effective reduction in the number of CD8+ and CD4+ T cells of PBMCs (peripheral blood mononuclear cells) of infected individuals can be obtained, which also can lead to impaired T memory cell formation and progression in the recovered SARS-CoV-2 patients (Kumar et al., 2020).

The antibody-mediated humoral immune mechanism occurs through the differentiation of B lymphocytes into plasma cells through T helper cells and the production of neutralizing antibodies specific for viral antigen (Kumar et al., 2020). The relevant virus-specific antibodies produced by B lymphocytes to limit SARS-CoV-2 infection are effective in preventing the entry of the virus to host cells. Thus, the specific antibodies prevent the transmission and recurrence of the infection by providing protection against the disease. SARS-CoV-2 S proteins have B cell epitopes as receptor binding domains (RBD) to produce neutralizing antibodies. Current evidence suggests that the Th1-type response is important in controlling SARS-CoV and this probably also applies to SARS-CoV-2 (Kumar et al., 2020).

In the literature review, it was found that in cases that result in death after SARS-CoV-2 infection, Th2 increases the pathogenesis of the virus with increased immune response (Huang et al., 2020; Zhou and Zhao, 2020). Similarly, it has been concluded in many studies that the triggering of the Th1 immune response plays a key role in a suitable vaccine model to be developed against the viral infections (Channappanavar et al., 2014; Huang et al., 2020; Thiel and Weber, 2008; Zhou and Zhao, 2020).

IFNγ mediated antiviral immune responses play a stronger cytokine role for control of SARS-CoV-2 infections. It has stated that low or delayed induction of IFNγ by the virus causes activation of macrophage and proinflammatory cytokines in the lungs. Activation of immune system components, cytokines or macrophages, causes vascular leakage as well as disrupting adaptive immune response (Kumar et al., 2020).

In conclusion it is vital to develop a vaccine against COVID-19 to prevent the disease and limit the outbreak, which has become a global disaster in 2020. Many different strategies are being examined to develop potential vaccines against the disease. Among these vaccines, the peptide-based vaccine strategies against SARS-CoV-2 have been mentioned in this review. Epitope-based peptide vaccines ascend among all the vaccines depending on their low costs, easier production and safety. However, there are some challenges to be overcome in developing an epitope-based peptide vaccine against SARS-CoV-2. Although in silico methods can predict the antigenic epitopes with high accuracy, it is probable that cell and animal studies may reveal low immunogenicity of the sequence. Therefore, a comprehensive study should be accomplished to predict the antigenic peptide sequence against the pathogen. Also, possible mutations in antigenic epitopes should be followed to keep the activity of vaccine formulation in the following batches of production. Low immunogenicity of the peptides compared to other vaccine types is the most significant drawback, which is overcome by using adjuvants in formulation. Proper selection of the adjuvant is essential in epitope-based peptide vaccines against SARS-CoV-2 to obtain a balanced Th1/Th2 immune response which will prevent immunopathology and antibody-dependent enhancement. In conclusion, it is evident that the epitope-based peptide vaccines have a great potential and should have a place in the competition for rapid development of a protective vaccine against SARS-CoV-2.

## Authors’ contribution

MT, ESA, TAr, DT-B, SD were responsible for the concept, design, and critical review of manuscript. Reviews: DT-B and EU in silico analyses; SD vaccine strategies; TAc, PPA, BU, and SD peptide synthesis and epitope-based peptide vaccine; MT adjuvants; and TAr and ESA immune response.
